# Dry under water: air retaining properties of large-scale elastomer foils covered with mushroom-shaped surface microstructures

**DOI:** 10.3762/bjnano.13.113

**Published:** 2022-11-21

**Authors:** Matthias Mail, Stefan Walheim, Thomas Schimmel, Wilhelm Barthlott, Stanislav N Gorb, Lars Heepe

**Affiliations:** 1 Nees Institute for Biodiversity of Plants, University of Bonn, Venusbergweg 22, D-53115 Bonn, Germany; 2 Institute of Nanotechnology (INT) and Karlsruhe Nano Micro Facility (KNMF), Karlsruhe Institute of Technology (KIT), Hermann-von-Helmholtz-Platz 1, D-76344 Eggenstein-Leopoldshafen, Germany; 3 Department of Functional Morphology and Biomechanics, Institute of Zoology, Christian-Albrechts-University of Kiel, Am Botanischen Garten 1–9, D-24118 Kiel, Germany; 4 Gottlieb Binder GmbH & Co KG, Bahnhofstr. 19, D-71088 Holzgerlingen, Germany

**Keywords:** adhesive tape, air layer, air retention, bionics, fouling, gecko tape, mushroom structures, passive air lubrication, Salvinia effect, superhydrophobicity

## Abstract

Superhydrophobic surfaces are well known for most different functions in plants, animals, and thus for biomimetic technical applications. Beside the Lotus Effect, one of their features with great technical, economic and ecologic potential is the Salvinia Effect, the capability to keep a stable air layer when submerged under water. Such air layers are of great importance, e.g., for drag reduction (passive air lubrication), antifouling, sensor applications or oil–water separation. Some biological models, e.g., the floating fern *Salvinia* or the backswimmer *Notonecta*, show long term stable air retention even under hydrodynamic conditions. Therefore, they are ideal models for the development of technical biomimetic air retaining surfaces. Up to now, several prototypes of such surfaces have been developed, but none provides both, stable air retention and cost effective large scale production. Meanwhile, a novel biomimetic surface is commercially available and produced on a large scale: an adhesive elastomeric film with mushroom-shaped surface microstructures that mimic the adhesion system of animals. In this study, we show that these films, which have been initially developed for a different purpose, due to their specific geometry at the microscale, are capable of stable air retention under water. We present first results concerning the capabilities of mushroom-shaped surface microstructures and show that this elastomer foil is able to stabilize a permanent air layer under water for more than two weeks. Further, the stability of the air layer under pressure was investigated and these results are compared with the predicted theoretical values for air retention of microstructured surfaces. Here, we could show that they fit to the theoretical predictions and that the biomimetic elastomer foil is a promising base for the development of an economically and efficient biomimetic air retaining surface for a broad range of technical applications.

## Introduction

**Figure 1:**
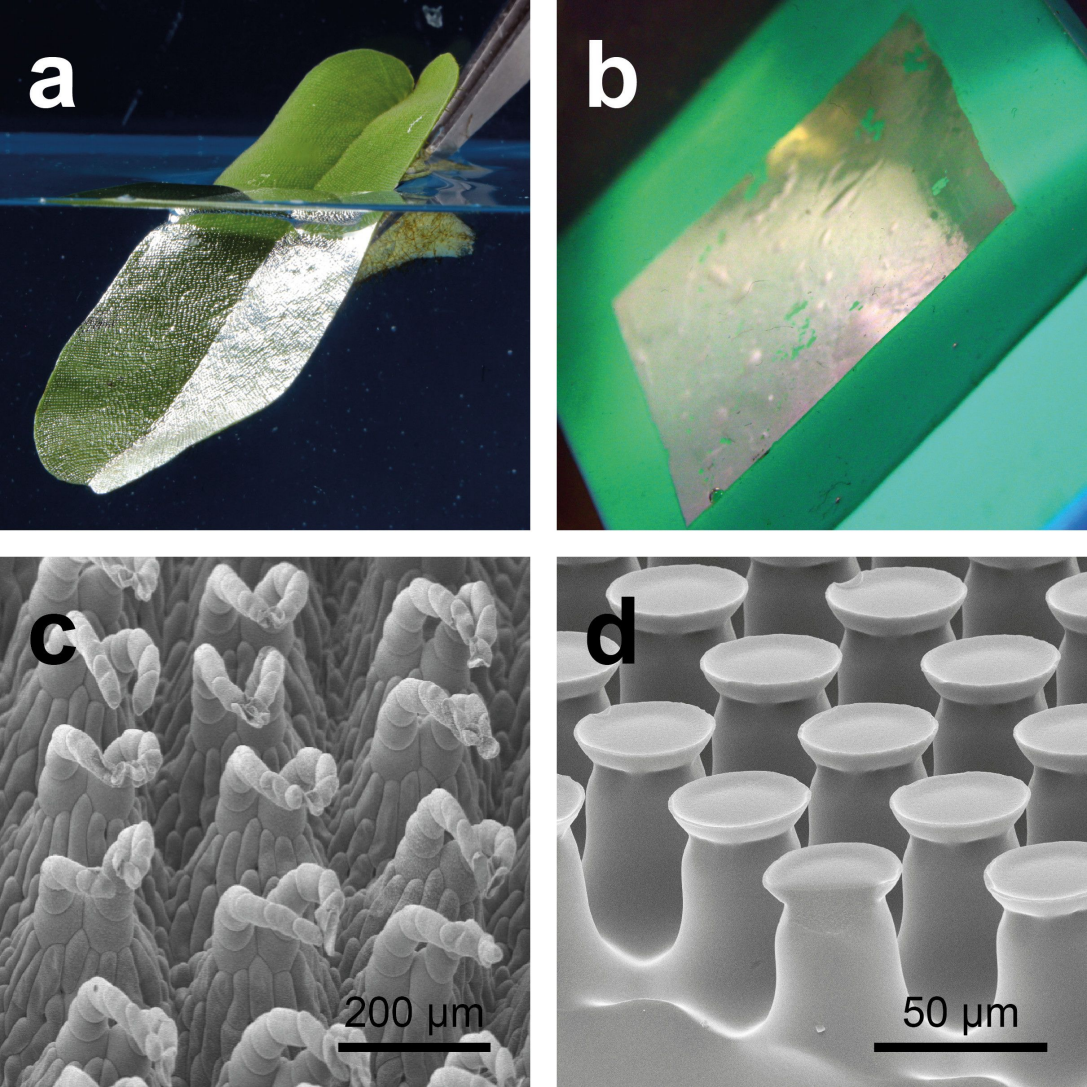
Biological role model and biomimetic air retaining surfaces. a) Leaf of the floating fern *Salvinia oblongifolia* submerged in water. The silvery shine indicates the kept air layer. b) Elastomer foil covered with mushroom-shaped surface microstructures (MSM) submerged in water. MSM-surfaces were glued to a green silicon surface to enhance contrast. Like on the biological role model, the silvery shine indicates the kept air. c) SEM image of the surface structure of the *Salvinia* leaf. d) SEM image of the MSM.

Superhydrophobicity is one of the key innovations in the biological evolution of organisms for the conquest of land [1]. Recently it was shown that this fascinating surface property evolved already in the cyanobacterium *Hassallia* [2] and is known for more than thousand years [3], but after the Lotus effect publication [4], this research led to a paradigm shift in surface science [5] and was the starting point for novel technologies in surface science [5]. Today many products in forms of coatings, sprays and paints providing superhydrophobic surfaces and self-cleaning properties are available on the market [1,6–7]. A most interesting feature of certain superhydrophobic surfaces is their ability to maintain a persistent air layer submerged under water. This ability is called Salvinia effect [5,8–10]. Due to the hydrophobic chemistry of the hierarchically structured surfaces water cannot penetrate and air remains trapped in between the structures [1], which is indicated by a silvery shine of the submerged surface (See Figure 1a). For technical applications, the Salvinia effect bears an immense potential, as air layers kept between water and a solid surface might serve as friction reduction agents, fouling protectors, corrosion protectors or for other applications, such as sensors [11–14].

Biological examples for such air retaining surfaces with most stable and persistent air layers were found on the floating ferns of the genus *Salvinia* (Figure 1a,c) and in the backswimmers *Notonecta* [1,15–16] and other aquatic organisms. In terms of technical applications, the Salvinia effect gained increasing interest in the past few years [1,3,9–10,13,15,17–25]. The most promising function for biomimetic air retaining surfaces is drag reduction. If an air layer is mounted between a solid surface and water flowing over this surface, the air layer serves as slip agent [26–28]. Such a drag reducing coverage allows significant friction reduction (up to 30%) in applications, where liquids flow over solid surfaces [29–30]. The highest economic and ecological potential for this technology is the shipping industry [31]. The optimal parameters for stable air retention have been previously investigated [1,32] and theoretical calculations have been performed [33–35]. Four criteria for the development of biomimetic air retaining surfaces have been found [1].

Transferring these parameters into technical surfaces, different prototypes have been developed, showing air retention over several years and drag reduction values up to 30% [24,36]. Up to now, all these prototypes are only at the laboratory scale and for different reasons difficult to realize in large-scale industrial productions. Here, a new and promising surface type is elastomer foils covered with mushroom-shaped surface microstructures (MSM) is introduced. These surfaces originate from the development of biomimetic adhesion systems. They are inspired by the feet of beetles, flies, spiders and geckos and have been shown to strongly enhance adhesion [37–51]. But these surfaces were also shown to be the structure of choice to produce omniphobic surfaces, their wetting behavior has been also previously described [52–53]. As these surfaces showed very good air retention properties, some of their structural parameters are in good accordance with some biological air retaining surfaces, and as they can be produced rather cost effective and on large scale, they could also be a promising base for the further development of biomimetic air retaining surfaces (Figure 1b,d). Further, this is a very nice example for the multifunctionality of some biomimetic-structured surfaces, showing that in nature often almost similar structures have evolved for completely different tasks. Here, we investigate the air retention capabilities of MSM for the first time. We analyzed the long term stability of the kept air layers as well as the behavior of the air layers at different pressures.

## Materials and Methods

### 1 Mushroom-shaped microstructured elastomer foil

Microstructured elastomer foils were made from poly(vinylsiloxane), a silicone elastomer. The surface has about 30,000 hexagonally distributed mushroom-shaped surface microstructures (MSM) per square centimeter (Figure 1d) [37–44]. Individual elements have a height of about *h* = 60 µm, an effective radius of about *r* = 15 µm, and a lattice constant *a* = 62 µm.

### 2 Persistence of air layers attached to microstructured surfaces immersed in water

Following the theory presented by Konrad et al. [33], *h*_max_ is the maximum persistence depth of an air layer attached to a microstructured surface immersed in water, i.e., for immersion depth below *h*_max_ the air layer is persistent and does not vanish, above it will disappear in time τ. All following calculations were performed for a temperature of 20 °C and an atmospheric pressure *p*_atm_ = 1 bar. The maximum persistence depth is given by


[1] $$h_{\max}=\frac{\sigma \cos\theta \left|\hat{\Sigma }\right|}{\rho_{\text{w}}g\left|\Omega \right|}\approx 2\text{ cm,}$$

whereas σ = 72.75 mN/m is the surface tension of water, cos θ is the water contact angle of poly(vinylsiloxane) which was assumed to be 95°, 
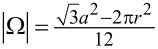
 and 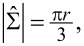 ρ_w_ = 997 kg/m^3^ the density of water, and *g* the gravitational acceleration.

The reduced bubble lifetime for *h* > *h*_max_


[2] $$\tau *=\frac{\tau \varepsilon }{V_{\text{i}}}\text{=}\frac{k_{\text{H}}}{4\pi D_{\text{a}}RT}\left(1+\frac{p_{\text{atm}}}{\rho_{\text{w}}g\left(h-h_{\max}\right)}\right)\text{,}$$

whereas *V*_i_ is the initial volume of the air layer, 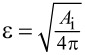 with *A*_i_ the interface area of *V*_i_, *k*_H_ is the Henry’s law volatility constant of air in water. In order to calculate *k*_H_ we assume air is composed of approx. 78% nitrogen and approx. 20% oxygen. Following Sander [54], the Henry’s law solubility constant for oxygen in water at 20 °C is approx. 1.42 × 10^−5^ mol/(m^3^·Pa), for nitrogen it is approx. 6.90 × 10^−6^ mol/(m^3^·Pa). Equating Henry’s law volatility constant for air, one gets approx. 1.27 m^3^·Pa/mol. *D*_a_ is the diffusion constant of air in water which is 2.5 × 10^−5^ cm^2^/s [55], *R* is the gas constant and *T* the absolute temperature.

### 3 Underwater air retention and its persistence

To demonstrate the underwater air retention and to analyze the stability of air layers kept by MSM, different samples were submerged in five different immersion depths, here 0.5, 5, 10, 15 and 20 cm. As a measure of the stability in dependence on the immersion depth the lifetime of the air layers were determined. Deionized water was used throughout all experiments.

#### 3.1 Air–water interface of a persistent air-layer

**Figure 2:**
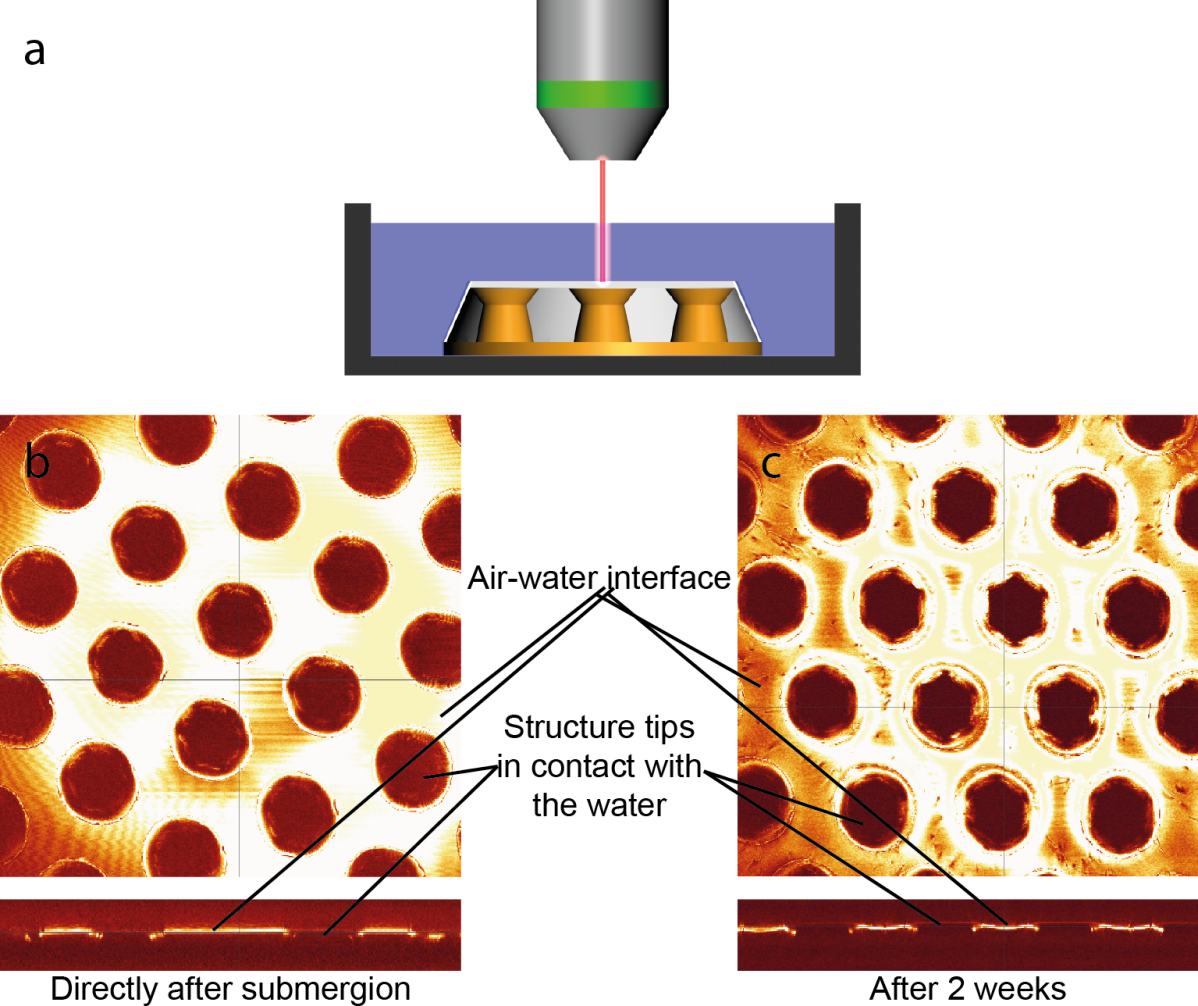
Confirmation of the persistence of the air layer in low water depth and analysis of the shape of the air–water interface by confocal laser scanning microscopy (CLSM) investigations. a) Schematic drawing of the principle of the measurement. The sample is submerged under water and keeps an air layer. The laser beam of the CLSM is reflected at the air–water interface. This enables the 3 dimensional analysis of the air layer. b) Result of the measurement directly after submerging the sample in 5 mm water depth. A top view image as well as a cross section of the air–water interface is shown. The dark parts in the top view image represent the MSM, the bright parts show the air–water interface in between. Also in the cross section the air–water interface is represented by the bright areas of the image while the MSM in between appear darker. It could be seen that the shape of the air layer is almost completely flat. c) Results of the measurement of the same sample after 2 weeks under water. Still the air–water interface is spanned between the tips of the MSM. The cross section shows almost no deformation of the air layer, indicating that almost no air is lost and the system is in an equilibrium state.

To investigate the shape of the air–water interface of the air layers kept by MSM and to proof the expected durability of the air layer in a water depth lower than 2 cm, a 2.0 × 2.0 cm^2^ sample was placed in a Petri dish filled with deionized water. In this first experiment, the air layer of the sample immersed at 0.5 cm depth, i.e., smaller than *h*_max_, was visualized with a confocal laser scanning microscope (CLSM, TCS SP II, Leica Microsystems, Wetzlar) using an objective lens (HCX APO L 63x/0.90 W U-V-I, Leica Microsystems, Wetzlar) with 63-fold magnification directly after submergence and after two weeks. Using the total reflection of the laser light at the air–water interface this method allowed us to analyze the shape of the air–water interface at high resolution. The method as well as the results are shown in Figure 2. For the analysis of the shape of the air–water interface, cross sections trough the image stacks have been generated by using the software Leica TFS.

#### 3.2 Static lifetime tests of air layer submerged deeper than *h*_max_

In a second experiment, which was done in two different laboratories, the air layer of samples placed at 5, 10, and 15 cm immersion depth, respectively and at 5, 10, and 20 cm immersion depth, respectively, i.e., larger than *h*_max_ were analyzed.

In one case, in each depth (5, 10, and 15 cm) two samples with a size of 2 × 2 cm^2^ have been placed. The semi-transparent MSM surfaces were glued to a green silicon surface to enhance contrast (air layer/wet surface). The samples were photographed using a digital camera (EOS-650D, Canon, Krefeld) every 3 min until the samples were completely wetted and no retaining air was visible. The area of the silvery shining air layer covering MSM was measured using Photoshop CS6 (Adobe Systems Software, Dublin, Ireland).

Also for the second set of samples (depth 5, 10, and 20 cm) a camera (PX-8085-919, Somicon^®^, Pearl GmbH, Buggingen, Germany) was used to take time series of images, which then where analyzed automatically using a self-written software tool based on the platform processing in Java.

**Figure 3:**
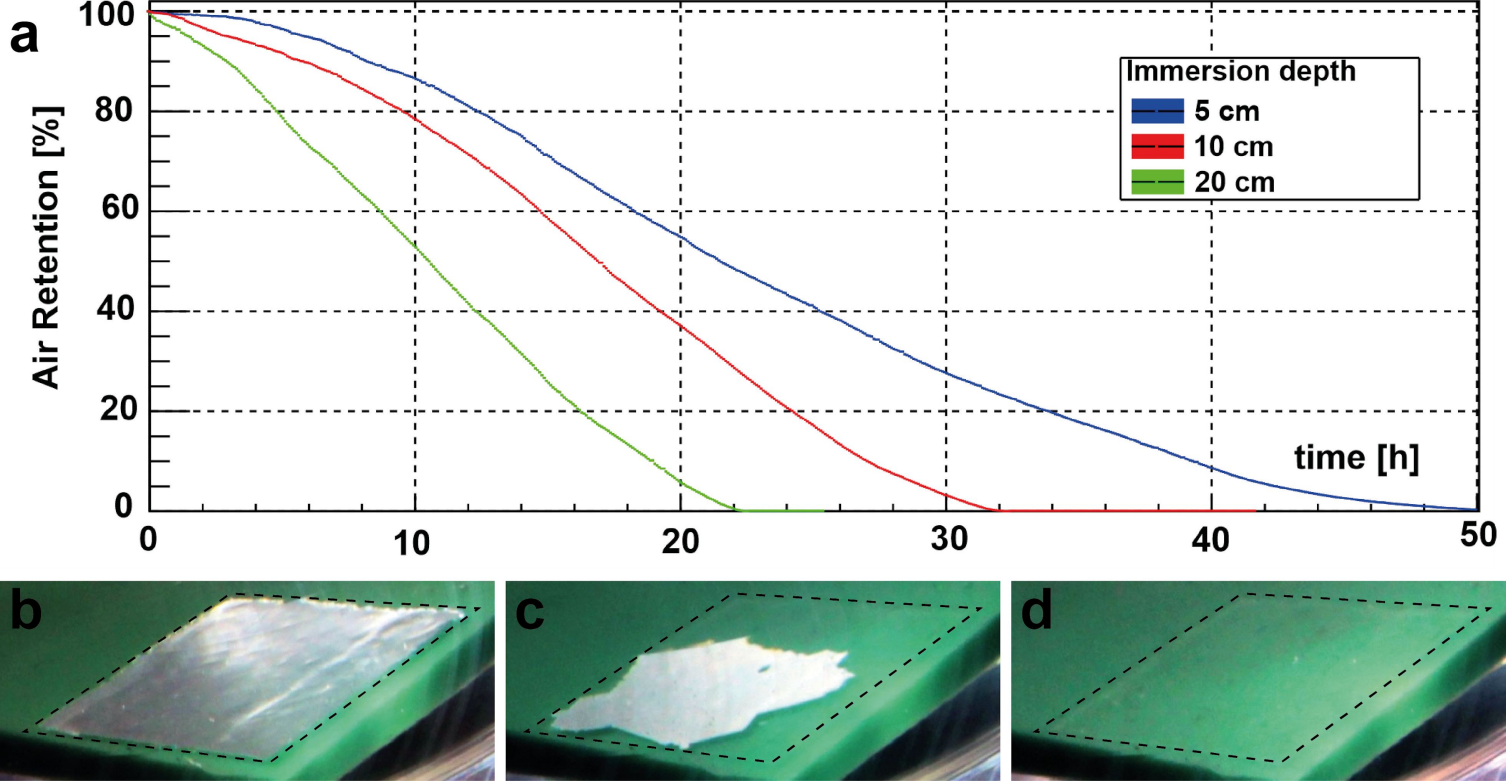
Results of the long term investigations of air layers on MSM in three different depths. a) The graphs show the sample area covered by an air layer in three different depths (5, 10 and 20 cm) over time. b–d) Examples of the images taken in the experiment. MSM surfaces were glued to a green silicon surface to enhance contrast, and imaged in defined time steps. b) At the beginning of the experiment, the sample (indicated by a dotted line) was completely covered by air. c) After several hours under water, part of the layer was lost. d) Finally, all the air was lost and the sample was completely wetted.

In Figure 3b–d examples of the images taken of a sample at different times are shown. Figure 3a exemplarily shows the measured time curves of one of the setups (depth 5, 10, and 20 cm). This method allows no quantitative analysis, but as the sample images and the curves in Figure 3 indicate, the air layers showed the expected behavior and disappeared faster in cases where the samples were placed deeper in the water as in those samples placed closer to the water surface.

#### 3.3 Simulation of static durability for immersion depth much deeper than *h*_max_

**Figure 4:**
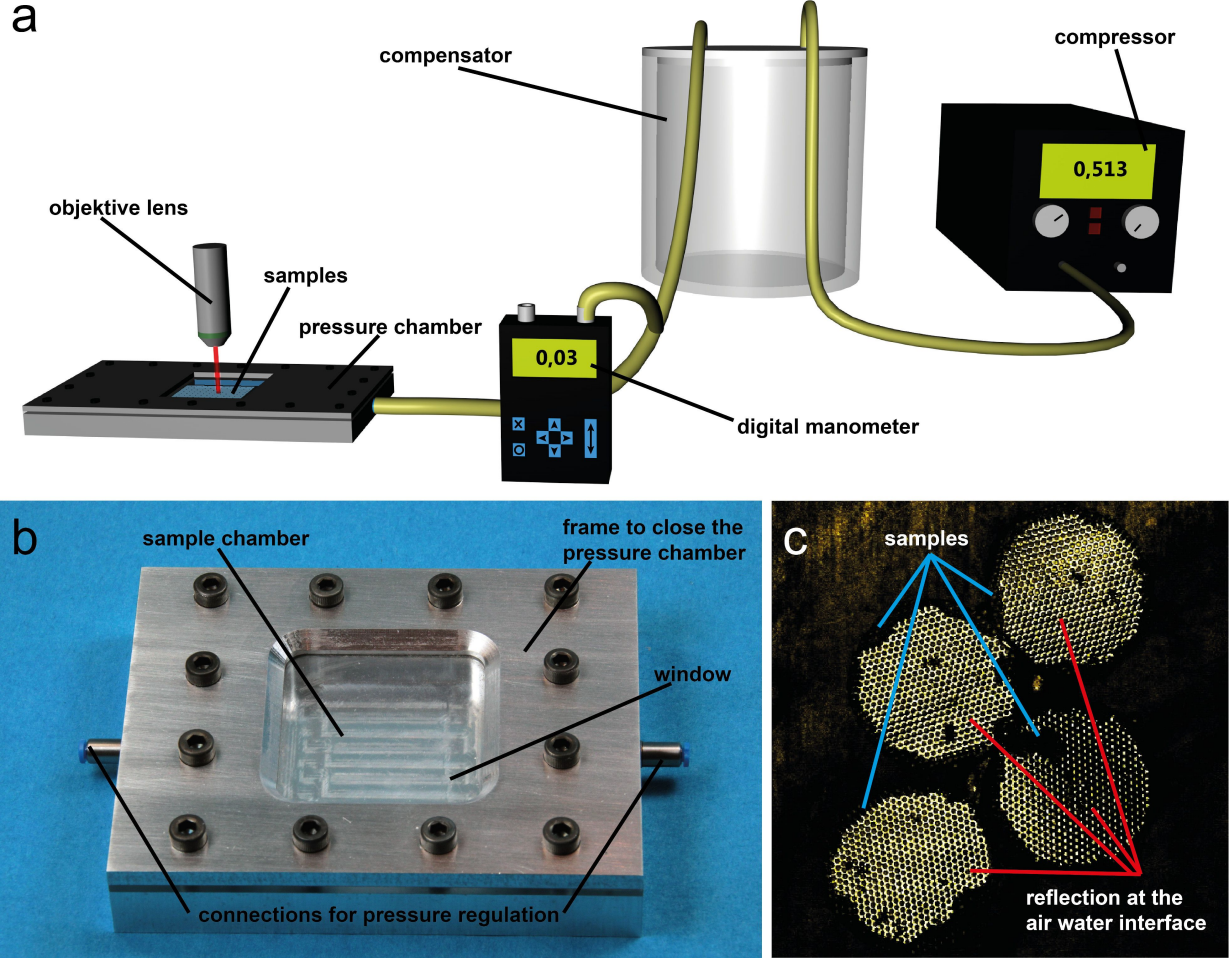
To determine pressure stability and diffusion behavior of the MSM, CLSM and a custom-made pressure chamber were used. a) Schematic drawing of the experimental setup, described in the experimental section. b) Photo of the custom-made pressure chamber. c) Examples of the images taken with the CLSM to investigate pressure and diffusion behavior. The bright areas on the four rounded samples in the image show the surface parts which keep an air layer and therefore show total reflection at the air–water interface. These areas have been measured and their size has been monitored over time, to analyze the changes at different pressure loads.

In order to simulate even greater immersion depth of the order of several meters the air layer kept by the microstructured elastomer foil was analyzed within a home-build pressure chamber. The setup is shown in Figure 4. The pressure chamber was connected to a compressor. To avoid pressure fluctuations, a compensator has been mounted between the pressure chamber and the compressor. Further a digital manometer has been connected to the pressure chamber to control the pressure in the sample chamber. With a valve the pressure in the chamber was regulated. Four circular samples with a diameter of 1.5 mm were placed simultaneously inside the pressure chamber. Then, the chamber was flooded with deionized water. The chamber was placed under a Zeiss LSM700 confocal laser scanning microscope system (Carl Zeiss Microscopy GmbH, Jena, Germany). The air–water interface of the samples was observed while the pressure was increased and kept at a certain level. Imaging was continued until all the air disappeared. First, a pressure of 500 mbar was applied corresponding to an immersion depth of about 5.1 m. Then the same procedure was performed with an applied pressure of 1000 mbar corresponding to an immersion depth of about 10.2 m. Analysis of the air covered areas was performed using Photoshop CS3 (Adobe Systems Software, Dublin, Ireland).

### 4 Calculation of the air layer volumes

From the measured areas of air layers covering the microstructured elastomer foils, the corresponding air volumes were estimated as follows:


[3] $$V=Ah\left(1-\rho \pi r^{2}\right)$$

with *A* the measured areas of air layers, *h* the height of a surface microstructure, *r* the effective radius of a surface microstructure, and ρ the number of surface microstructures per unit area which was assumed to 30,000 per cm^2^.

Upon hydrostatic pressure, either applied by immersion depth or by an applied pressure (see sections 3.2 and 3.3), an initial air layer with volume *V*_0_ will reduce to the volume *V*_1_ by


[4] $$V_{1}=\frac{p_{0}V_{0}}{p_{1}+p_{0}}.$$

### 5 Long term storage

MSM samples were submerged in a jam jar filled with tap water to a height of about 1.5 cm, i.e., below *h*_max_ to allow for a persistent air layer. The jar was closed and stored on a rack in the office. After one month, the samples were taken out of the water and dried in air. The dried samples were mounted to aluminum stubs and fixated using silver paste. To enhance the conductivity, the samples were sputtered with a thin gold layer (thickness 20 nm, Sputter Coater 108 auto, Cressington, Dortmund). Afterwards the samples have been analyzed using SEM (15 kV, CAMBRIDGE Stereoscan 200 SEM, Zeiss AG, Oberkochen).

## Results and Discussion

As a first step of the investigation of the air retaining capabilities of mushroom-shaped surface microstructures (MSM) the long-term stability of the air layers was analyzed. Calculations according to Konrad et al. [33] predicted a maximum immersion depth underwater *h*_max_ of about 2 cm for a persistent air layer kept by MSM with the given geometric dimensions and the given surface energy (see section 2). If they are submerged deeper than 2 cm, the air layer gets lost over time due to diffusion effects.

To confirm the predicted persistence of the air layer in a water depth below 2 cm samples have been submerged in a depth of about 5 mm for two weeks. The analysis of these samples by confocal laser scanning microscopy (CLSM) showed the expected persistence of the air layer (see Figure 2). The images taken directly after the submersion of the sample show an almost perfectly flat air–water interface. After two weeks under water the same sample still kept an air layer and was analyzed again by CLSM. In Figure 2 it could be seen that the air–water interface showed only a slight indentation between the MSM. This is due to a little loss of air which happens before air layer and water were in an equilibrium state which from then on was stable over the entire time period. This result is in good agreement with the theoretical predictions of Konrad et al. [33] and shows the long term stable air retention capabilities of the MSM under this shallow water conditions.

Tests with samples immersed in depths larger than *h*_max_, i.e., 5, 10, 15 and 20 cm showed the expected vanishing of the air layer over time. With higher immersion depth the faster the air got lost (see Figure 3). The time it took till the entire air was lost is in good accordance with the predicted time calculated with the methods of Konrad et al. [33].

To further investigate the stability of the air layers kept by MSM and to analyze their pressure and diffusion behavior, measurements under static pressure in a custom-made pressure chamber and again by using CLSM were carried out. The principle of these measurements is described in the experimental section and shown in Figure 4. By applying a constant pressure a deeper submersion of the samples was simulated. As expected, it turned out that the higher the pressure, the faster the decrease of the air layers. The first reduction of the air covered area on the surfaces was due to the compression of the air under pressure. This compression of the air led to a lateral decrease in the area covered by air, leaving an unchanged high layer of air between the MSM structures. This lateral "melting" of the air layer patch then continued by diffusion of the air into the water until finally no air was visible.

**Figure 5:**
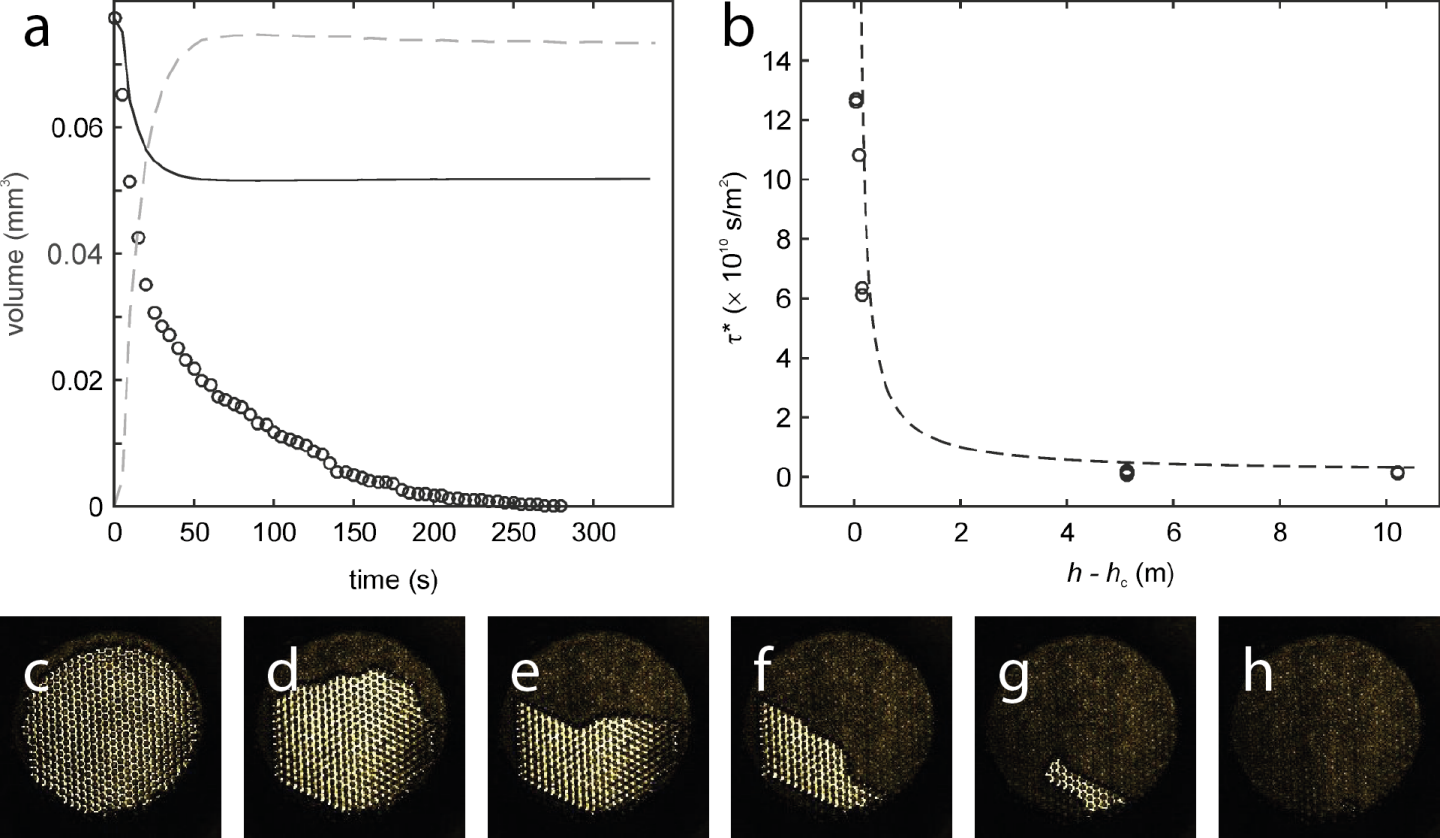
Results of the pressure stability and diffusion behavior experiments. a) With increasing pressure (dashed line), the air volume gets compressed. After calculation of the theoretical compression of the air volume (continuous line) and the decreasing of the air covered surface area due to this compression it was possible to calculate the loss of air due to diffusion over time (circles). The results are in good accordance with the predicted behavior after Konrad et al. [33]. b) Comparison of measured results with theoretical predictions. The dashed curve shows the theoretically predicted life time of the air layer on a submerged MSM surface depending on the immersion depth, calculated after Konrad et al. [33]. The various circles represent the results of the long term stability tests and the pressure stability and diffusion behavior experiments. The results are in good accordance with the predicted values. c–h) Examples of the images used for these calculations. The images show the decrease of an air layer on a sample in a time span of 6 min at pressure of 1 bar. c) Initial state directly after closing the pressure chamber. Almost the entire sample surface is covered by a reflecting air layer. d–g) With increasing pressure and due to diffusion the air gets lost. h) After 6 min the entire air was lost.

After elimination of the volume loss due to compression of the air layer, the loss of air due to diffusion can be quantified. The graphs of these measurements show the expected progress (see Figure 5).

For comparison and validation, the theoretical lifetime of the air layer at different pressures/depth and maximum depth for persistent air retention on MSM has been calculated, using the structural parameters of the MSM and the equations of Konrad et al. [33] (see sections 2 and 4). The result is shown in Figure 5. The maximum depth for permanent air retention was about 2 cm. Further, the results of the stability tests at different depth and the investigations of the diffusion behavior at different pressures are plotted in the graph. The results are in good agreement with the theoretically predicted values.

Further, our results indicate that both the size and shape of the contact area between water and air plays a crucial role for the stability of the air layer. The loss of air happened not only at the interface of water and air between the top side of the MSM, but also on the sides. This means the water-contact-area/volume ratio of the air layer is a crucial parameter for the long term stability of the kept air. Previous works have focused primarily on the behavior of the air–water interface at the tips of the surface structures. However, the sides also play a decisive role. Assuming a round surface section, the air volume resembles a cylinder. Thus, there is not only contact between air and water at the top surface structures, but also at the shell surface, through which additional diffusion takes place. Therefore, the surface should be compartmented to exclude the influence of these side surfaces. Furthermore, a subdivision of the air volume into small individual volumes ensures better stability, since individual defects only affect a partial volume and not the total volume. This once more indicates that compartmentation of air retaining surfaces is of great importance, as this would lead to much more stable air retention [56].

Beside the investigation of the air layers kept by submerged MSM in deionized water and the comparison with the theoretically predicted values, also the SEM investigation of the samples submerged for one month in tap water offered some important results for the practical application of air retaining MSM.

Mushroom-like microstructures are known for their antifouling properties against such hard-foulers as barnacles [53,57–59]. However, in air–water boundaries under non-sterile and static conditions an „interphase microbial community“ (neuston) usually becomes established in fresh- and saltwater – like acetobacter and yeast communities in the production of vinegar [60].

**Figure 6:**
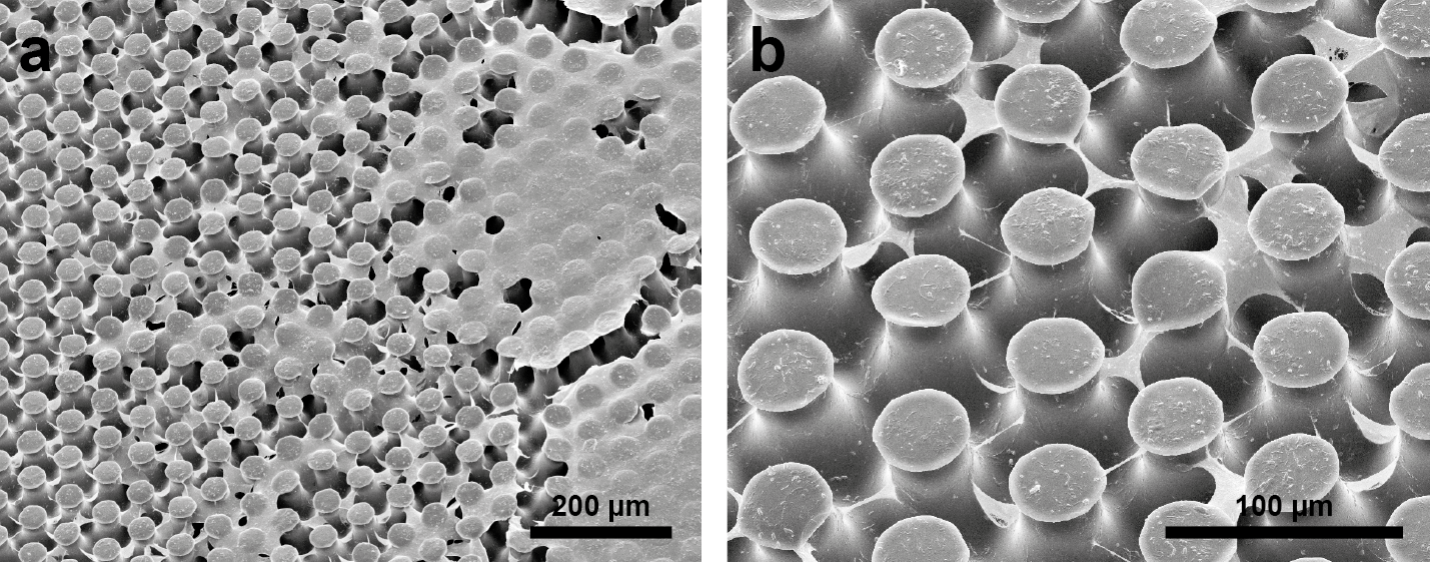
SEM images of the MSM after they have been submerged for one month in tap water. a) A microbial neustonic on the MSM. The biofilm covers large areas of the MSM surface. b) Already after one month the tips of some of the MSM are contaminated by bacteria and connected by straight filaments, which are most probably fungal hyphae.

Since the air layers on some Salvinia-like biomimetic surfaces are permanent over years, like in many air–water interfaces a neustonic microbial biofilm (“Bacterioneuston”), usually associated with fungi, becomes established under non-sterile and non-turbulent conditions rather fast. On the MSM already after one month the air–water interface is contaminated by bacteria and the tips of the MSM are contaminated by bacteria and connected by straight filaments which probably represent fungal hyphae (see Figure 6, compare, e.g., illustrations in [61]). Under hydrodynamic conditions this microbial fouling effect can probably be neglected and thus does not hinder the application of MSM under turbulent conditions.

## Conclusion

In this work the air retaining properties of large-scale elastomer foils covered with mushroom-shaped surface microstructures (MSM) have been analyzed for the first time. It has been shown that these surfaces are able to keep stable air layers under water for more than two weeks, but, due to the morphology of the surface a permanent stabilization of air is only possible to maximum depth of about 2 cm. The theoretical predictions of Konrad et al. [33] were confirmed. These results and the fact, that MSM are comparatively cheap and easy to produce on large scales, show, that MSM are a promising new alternative for the development of biomimetic under water air retaining surfaces. However, as other prototypes, they show stable air retention in low depth but lose their air layers in higher depth due to diffusion of the air into the water. For a use of their air retention capabilities in technical applications in higher water depths and under real conditions, a method to refill the air layer after a loss of air occurred have to be found.
